# Resonant Soft X-ray Scattering for Organic Photovoltaics

**DOI:** 10.1021/acs.jpcb.5c00362

**Published:** 2025-03-26

**Authors:** Dean M. DeLongchamp

**Affiliations:** Materials Science and Engineering Division, National Institute of Standards and Technology, 100 Bureau Drive, Gaithersburg, Maryland 20899, United States

## Abstract

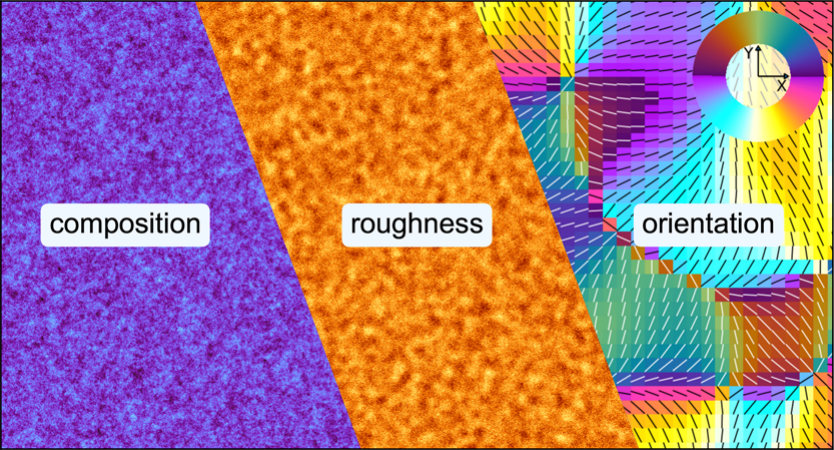

Resonant Soft X-ray Scattering (RSoXS) has emerged as a powerful technique for probing the morphology in organic bulk heterojunction (BHJ) solar cells, frequently employed as a measurement of phase purity and compositional length scales. Here we use the National Institute of Standards and Technology RSoXS Simulation Suite to systematically examine how structural features common to BHJs would contribute to RSoXS patterns in the PM6:Y6 BHJ system. Starting from experimentally determined anisotropic optical constants, we simulate scattering from controlled morphological variations including compositional heterogeneity, interfacial sharpness, surface roughness, and molecular orientation. Our results demonstrate that noncompositional features can cause increases in scattering intensity exceeding those from compositional phase separation. Surface roughness of just a few nanometers produces substantial scattering due to the high contrast between organic materials and vacuum, and molecular orientation effects—whether random, interface-aligned, or independently correlated—can dramatically influence pattern intensity and shape. However, each structural feature exhibits a distinct energy-dependent scattering signature across the carbon K-edge, suggesting that careful analysis of the complete spectral response could enable deconvolution of multiple contributions. These findings provide a broader interpretation of the excellent correlations between RSoXS measurements and BHJ solar cell device performance, while highlighting the potential of forward simulation approaches to leverage the full information content of energy-dependent RSoXS measurements.

## Introduction

Organic photovoltaics (OPVs)^1,2^ are a promising candidate technology for sustainable, lightweight, and flexible solar energy conversion. Solution-processable OPVs typically use an active layer structured as a bulk heterojunction (BHJ),^3,4^ a nanoscale interpenetrating network of donor and acceptor organic semiconductors. The spatial arrangement and phase purity of these components are thought to be critical for efficient photon-to-electron conversion. Upon light absorption, excitons—bound electron–hole pairs—are generated and must diffuse to a heterojunction interface within their finite diffusion length, typically on the order of ≈10 nm,^5^ to dissociate into free charges. High phase purities^6−9^ and percolative, cocontinuous domains^10−12^ are thought to facilitate continuous charge transport pathways and minimize exciton recombination. For effective exciton dissociation, the characteristic length scales of the BHJ morphology must be comparable to or smaller than the exciton diffusion length, ensuring that exciton generation sites are always within reach of a heterojunction interface. Less pure phases can lead to increased recombination events and the formation of trap states that hinder charge transport,^13^ thereby reducing photocurrent and overall device efficiency. On the other hand, some amount of phase mixing may be required to maintain facile charge carrier transport.^14^ Precise optimization of BHJ morphology and phase purity is therefore paramount for enhancing exciton diffusion, charge separation, and transport processes, driving OPV technologies toward higher power conversion efficiencies (PCEs) and broader commercial viability. These considerations frame a key challenge to the OPV community: for morphology and phase purity in BHJ OPVs to be optimized, they first must be measured.

A host of experimental techniques have been developed and refined to measure different aspects of morphology in OPV BHJ films.^15−18^ Transmission electron microscopy (TEM)^19−21^ and atomic force microscopy (AFM)^6,22^ provide high-resolution images revealing surface and cross-sectional morphologies, including direct visualization of domain shape and size. These microscopies are inherently two-dimensional: TEM offers a projection through the film, whereas AFM measures topographical features at the surface. Three-dimensional composition-sensitive imaging modalities remain limited, though some promising approaches are growing in capability such as infrared-sensitive AFM modalities^23^ and TEM energy filter approaches in combination with tomography.^20^ Scattering techniques such as grazing-incidence small-angle X-ray scattering (GISAXS) and grazing-incidence wide-angle X-ray scattering (GIWAXS, also known as grazing-incidence X-ray diffraction^24^) readily capture three-dimensional structural information averaged over large volumes. GISAXS measures nanometer-scale domain shape and size,^25^ whereas GIWAXS can access unit cell dimensions, crystalline fractions, and crystallite orientation distributions. Among the reciprocal-space methods, resonant soft X-ray scattering (RSoXS)^26,27^ stands out for its ability to interrogate both compositional and orientational heterogeneities within BHJ systems. By exploiting the energy-dependent near-edge X-ray absorption fine structure (NEXAFS)^28−30^ of organic materials for enhanced contrast, RSoXS can achieve high chemical contrast between donor and acceptor phases, enabling quantification of phase purity at nanometer-length scales. RSoXS can also derive contrast from molecular orientation heterogeneity,^31,32^ a capability unique among small angle scattering (SAS) methods. The thin film sample requirement (≲250 nm) for RSoXS is particularly well-matched to the optimal film thicknesses for BHJ OPVs and other organic semiconductor films. Its unique combination of composition and orientation sensitivity makes RSoXS particularly well-suited to elucidate the complex interplay between morphology and optoelectronic properties in OPVs, and RSoXS has become a key component of multimodal strategies for comprehensive morphological characterization.^8,33−37^

Phase purity can be measured by many experimental techniques, although it is particularly challenging to do so in BHJ morphologies. As we will discuss more below, RSoXS measurements of phase purity are almost always *relative*, meaning that the absolute phase purity is not measured by the technique and comparisons can only be made to the “most pure” sample in a homologous series. Even with this limitation, RSoXS regularly reports differences in phase purity in homologous series that can range across factors of 1.1× to 5× depending on the system. Other techniques can be more quantitative, but typically not when measuring the BHJ morphology. A particularly robust platform that we recommend for fundamental miscibility studies is thin-film bilayer mixing experiments, the results of which can be probed by a variety of methods including neutron reflectivity, ellipsometry, secondary ion mass spectrometry, and cross-sectional electron microscopy techniques.^38−42^ These studies broadly demonstrate that significant variability in quasi-equilibrium phase purity is common in mixtures of organic electronics materials, with observations ranging from essentially complete immiscibility to essentially complete miscibility for various pairs of materials. We note that most of these careful miscibility studies apply to arrested thermal interdiffusion, and there is a general expectation that greater extents of mixing may be possible in solution-cast BHJ films, which have a greater potential to achieve nonequilibrium phase displays, and furthermore in which different processing choices for the same material combinations might result in dramatically different extents of phase mixing.

By an accident of history, the RSoXS technique has become strongly associated with organic electronics materials because the challenge of measuring OPV BHJ morphologies arose contemporaneously with the advent of RSoXS facility availability and the accompanying maturation of experimental approaches and analysis frameworks. The strong and accessible contrasts afforded by the varied 1s → π* manifolds of organic electronics materials lowered the threshold for application of RSoXS compared to other materials and further reinforced the appearance of a necessary connection. It is important to emphasize, however, that RSoXS is not confined to organic electronics materials; the technique enjoys broad application in soft matter studies and should be considered part of a SAS measurement continuum that includes conventional small-angle X-ray scattering (SAXS) and small angle neutron scattering (SANS), with RSoXS having unique contrast modalities for soft materials.^27^ Some of the earliest demonstrations of RSoXS focused on thin films of block polymers, where the compositional contrast offered by RSoXS enabled the separation of contributions from different block chemistries.^43,44^ RSoXS capabilities have been used more recently to study directed self-assembly of block polymers for lithographic applications^45^ and complex hierarchical triblock structures.^46^ RSoXS has also been applied to measure chain orientation in polymer-grafted nanoparticles,^31^ compositional separation in small-molecule glasses,^47^ RO membranes,^48^ ionomers,^49^ and even biological assemblies.^50^ Although we focus on the application of RSoXS to BHJ OPVs, the contrast concepts described here are universal and the conclusions are potentially broadly applicable to RSoXS measurements across all soft matter.

### Contrast Fundamentals: The Basis for Phase Purity from RSoXS

The contrast in RSoXS measurements arises from differences in the energy-dependent complex refractive index (*n*) between the donor and acceptor materials, expressed for each material as:
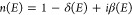
1where δ is the dispersive component and β is the absorptive component. The absorptive component β(*E*) directly corresponds to the NEXAFS spectrum; it exhibits pronounced peaks at specific energies associated with electronic transitions. The NEXAFS peaks in β(*E*) lead to significant inflections in δ(*E*) near the absorption edges due to Kramers–Kronig relations, which link the real and imaginary parts of the refractive index.^51^ The scattering contrast *C*(*E*) at a given energy *E* in a two-component system can be quantitatively described by:
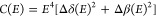
2where Δδ(*E*) and Δβ(*E*) are the differences in the dispersive and absorptive components of the refractive index between the two phases, respectively.^52^*C*(*E*) serves as a measure of phase purity because δ and β represent the effective refractive indices of each phase. To a first approximation, the X-ray refractive index of a mixed phase is a volume-fraction-weighted average of the constituent indices. Consequently, as the two phases become more mixed, the differences in δ and β diminish, leading to reduced scattering contrast. A key principle of RSoXS is to exploit contrast enhancements from excursions in δ and β that occur due to bond-specific NEXAFS excitations in the materials being measured; the contrast derived from these differences can exceed that in conventional SAXS and even SANS with expensive radiolabeling, by factors of 10× or more. Interestingly, the maximum contrast does not always align with the peak energies of β(*E*) (NEXAFS peaks). Instead, the energy of maximum contrast is often observed on the shoulders of these peaks because of inflections in δ(*E*) near the absorption edges.

The use of RSoXS contrast to measure phase purity then requires an approach to evaluate the effective contrast in an RSoXS experiment. Classic approaches to the measurement of relative or absolute contrast in the SAXS and SANS communities involve the Total Scattering Invariant (TSI), sometimes called simply the “invariant” or “Porod invariant”, and sometimes also referred to as “Total Scattering Intensity”. The TSI can be expressed as:
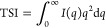
3where *I*(*q*) is the scattered intensity as a function of *q*, the magnitude of the scattering vector. The expression shown in eq 3 expects that *I*(*q*) is isotropic in all directions and that a measurable *I*(*q*) is available at every *q* value. In the limit where these assumptions are true, and for a two-component system with an energy-dependent contrast:
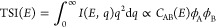
4where ϕ_A_ and ϕ_B_ are volume fractions of the two phases. Combining eqs 2 and 4, variations in  would be linearly proportional to variations in Δβ and Δδ, which reflect phase purity.

The practical application of the TSI concept to OPV BHJ purity measurement by RSoXS includes some significant approximations and assumptions. The RSoXS experiment has intrinsic limitations in its *q* range. Beamstop edge-scattering artifacts and limited sample–detector distances on available instruments limit the low *q* range, and the measurement can become very noisy at high q, with a fundamental high-*q* limitation in the diffraction limit which, for carbon K-edge RSoXS, is relatively low compared to other SAS methods at *q* ≈ 3 nm^–1^. Practical RSoXS experiments rarely collect patterns beyond *q* ≈ 1 nm^–1^ at the carbon K-edge. Additionally, *I*(*q*) for RSoXS of OPV BHJs is not expected to be isotropic, as is assumed in eq 4, but the typical RSoXS experiment only measures *I*(*q*) at 90° incidence, with scattering vectors roughly parallel to the sample plane. Significant crystal textures, and anisotropic morphological traits (fibrils, platelets, etc., that might be oriented parallel to the substrate) are observed in OPV BHJs and indeed most organic electronics films, and it is expected that scattering vectors orthogonal to the sample film plane might deliver different patterns and scattering intensities. These scattering vectors might be accessed to some extent by tilting the sample during RSoXS measurement, exploring incident angles other than 90°, but absorption typically occults the scattering pattern completely at incident angles shallower than ≈45°.

The RSoXS measurement of OPV BHJ phase purity therefore uses practically available RSoXS data to create an approximation of TSI. Proceeding from *I*(*q*) collected at 90° incidence, a similar integration is performed:
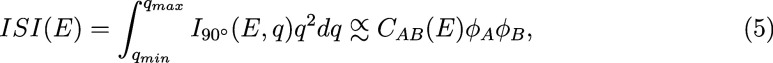
5where “integrated scattering intensity” (ISI) is used by many to acknowledge the approximations made and the distinction between this quantity and the classical TSI. Thusly integrated ISIs are typically compared across a homologous series of differently prepared BHJs ostensibly to probe differences in phase purity. It is usually also assumed that ϕ_A_ and ϕ_B_ do not vary significantly across a homologous series, such that variations in  are interpreted as directly proportional to phase purity. A rationale for energy selection, if one is given, is most commonly to choose the energy of greatest resonant contrast enhancement.

This analysis approach, which we will hereafter refer to as the  analysis framework”, has emerged as a frequent analysis workflow for the RSoXS of OPV BHJs. Due to the volume of OPV research publications, it is also by far the most published RSoXS analysis framework. It is moreover a facile, model-free workflow that does not require interpretation of *I*(*q*) traces with respect to structural features, though some interpretation of *I*(*q*) is frequently provided. It has become common practice to assume that features in *I*(*q*) originate entirely from compositional heterogeneity between the BHJ components, and that variations in  faithfully reflect changes in phase purity.

### Challenging Common Assumptions in OPV BHJ RSoXS

Although RSoXS is widely employed for characterizing BHJ morphologies, these core assumptions—particularly that (1) phase purity is the dominant factor determining the ISI and (2) compositional heterogeneity exclusively governs the resonantly enhanced features observed in *I*(*q*) profiles—warrant careful re-examination. Here we will prove that factors such as molecular orientation heterogeneity and surface roughness can significantly influence RSoXS scattering intensity and scattering features. Trends in  with BHJ processing choices, molecular design, or other variables are just as likely - or more likely - to stem from monotonic variations in surface roughness or orientation heterogeneity. We will intentionally minimize citations to previously published applications of RSoXS in OPVs because the community is best served with an emphasis on correct approaches to the technique going forward. To explore these complexities, here we employ the NIST RSoXS Simulation Suite (NRSS)^53,54^ to generate realistic, energy-dependent scattering profiles describing BHJ films. We use experimentally measured anisotropic indices of refraction to describe a BHJ composed of the polymer electron donor PM6 and the nonfullerene acceptor (NFA) Y6, a high-power-conversion-efficiency model system first introduced in 2019^55^ that has become an important reference system in BHJ OPVs.^56−58^ By systematically varying realistic phase purity, surface roughness, and molecular orientation within our models, we quantify how each of these factors independently and collectively affects the ISI and contributes to the emergence of resonantly enhanced features in the *I*(*q*) traces. Our simulations reveal that variations in molecular orientation and surface roughness lead to changes in ISI of similar or greater magnitude to those originating from phase purity. An RSoXS experiment with a single photon energy cannot disentangle these contributions, but the energy dependence of each scenario is distinct.

These findings demonstrate how RSoXS is sensitive to a broader range of structural features than conventionally assumed. By disentangling the contributions of phase purity from those of orientation heterogeneity and surface roughness, our study underscores the necessity for comprehensive modeling and more careful interpretation of RSoXS data. This more nuanced approach enhances the utility of RSoXS, because it is more than a simple phase purity measurement. Used to its full potential, it can provide a previously unmeasurable, uniquely rich morphological description with which to develop correlations with power conversion efficiency and to optimize BHJ OPV performance.

## Experimental Methods

Anisotropic optical constants for PM6 and Y6 were taken from previously published angle-resolved NEXAFS measurements^59^ collected at 7-ID Spectroscopy Soft and Tender beamline^60,61^ at National Synchrotron Light Source (NSLS-II). Data were analyzed with QANT.^62^ All RSoXS simulations were performed using the NIST RSoXS Simulation Suite (NRSS) and confirmed working with recent version 2024.12.22.01, available at github.com/usnistgov/NRSS/. Calculations were executed on a small graphics processing unit (GPU) cluster equipped with three NVIDIA Quadro RTX 8000 graphics cards.^2^

The simulation volume consisted of a 1024 × 1024 × 128 voxel grid representing a 1.024 μm × 1.024 μm × 128 nm region of space. The BHJ was modeled as a 90 nm thick film (90 voxels) with 38 nm of vacuum above. Composition descriptions were generated using a Gaussian Random Field algorithm with specified correlation lengths. Surface roughness was implemented through height variations at the film-vacuum interface. Molecular orientation distributions were defined through either random assignment, composition gradient alignment, or independent correlation fields as described in the text. For each model configuration, RSoXS patterns were calculated at photon energies spanning the carbon K-edge. The  values were the result of integration from *q* = 0.01 nm^–1^ to *q* = 1 nm^–1^, a range similar to that of typical experimental RSoXS data.

## Results and Discussion

### Soft X-ray Indices of Refraction

**Figure 1:**
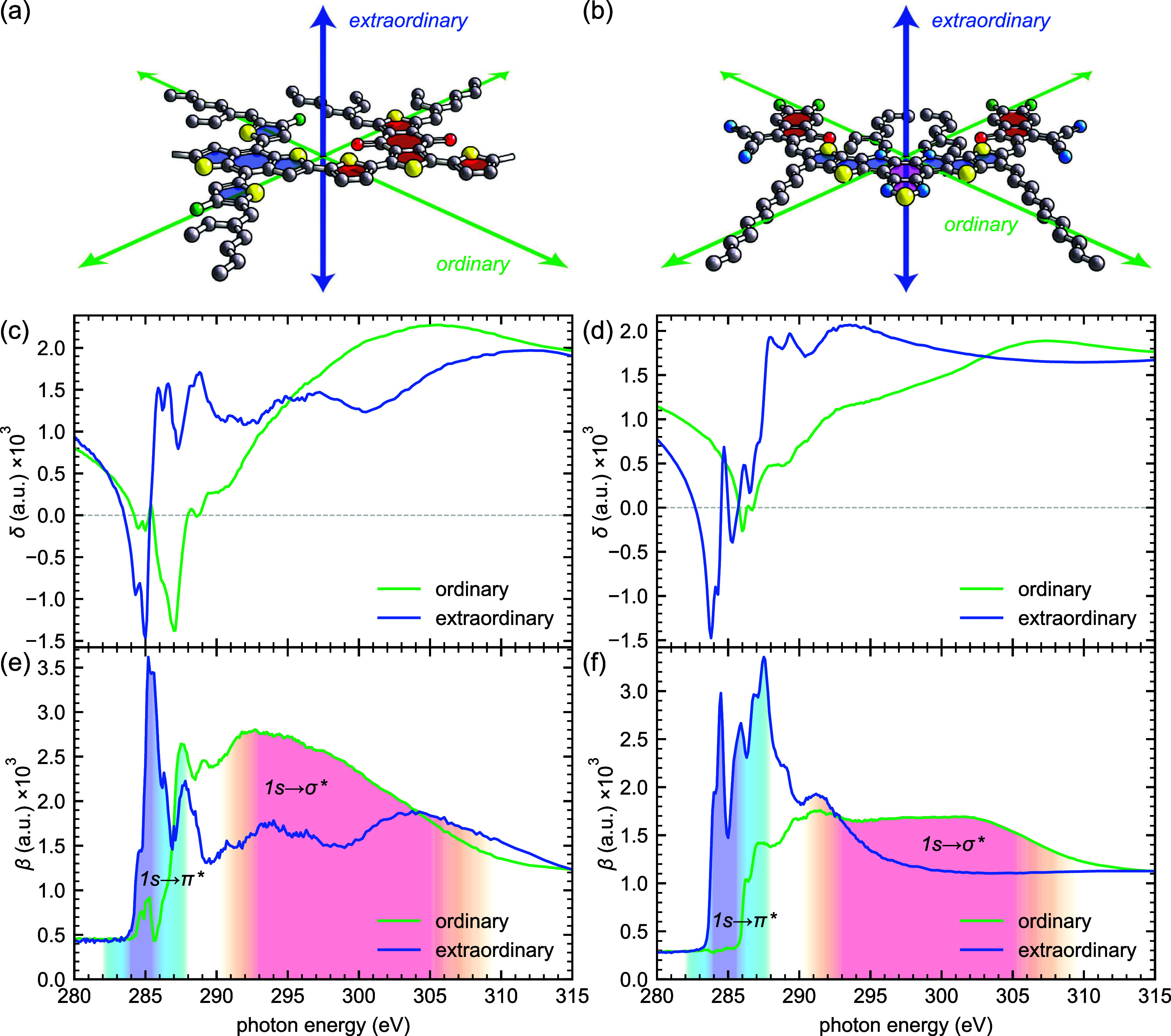
Complex refractive indices of PM6 and Y6 near the carbon K-edge. (a, b) Molecular structures of PM6 and Y6, respectively, with arrows indicating the extraordinary (blue, out-of-plane) and ordinary (green, in-plane) optical axes. (c, d) Dispersive component (δ) of the complex refractive index for PM6 and Y6, respectively. (e, f) Absorptive component (β) of the complex refractive index for PM6 and Y6, respectively, highlighting key electronic transitions. The 1s → π* transitions (blue shaded regions, 285 eV) and 1s → σ* transitions (red shaded regions, 290–305 eV) exhibit pronounced dichroism.

Accurate RSoXS modeling first requires an accurate description of the PM6 and Y6 complex refractive indices. Across the carbon K-edge, organic semiconductors exhibit strong optical anisotropy due to extensive π-conjugation, which can lead to coplanar ring systems and a characteristic common orientation of 1s → π* resonances. We use previously published NEXAFS spectra of PM6 and Y6^59^ to derive complex refractive indices across the carbon K-edge. For pure films of PM6 and Y6, partial electron yield (PEY) measurements were collected at several angles of the incident electric field vector with respect to sample normal. These angle-dependent spectra were analyzed to extract molecular frame absorbance along the extraordinary and ordinary directions as shown in Figure 1a,b, using difference spectra to separate the anisotropic response. The pure component Y6 film exhibited predominantly face-on orientation, whereas the PM6 film showed edge-on character. For PM6, an additional transformation was required wherein the extraordinary and ordinary axes were interchanged; this transformation assumes PM6 exhibits uniaxial optical behavior and that the NEXAFS response is similar for electric field vectors parallel to either the long or short axis of the conjugated plane. The molecular frame NEXAFS spectra were then scaled using atomic scattering factors, material density, and atomic composition to obtain β, from which δ was calculated using the Kramers–Kronig-consistent kkcalc Python package,^51,63^ with results shown in Figure 1c–f. An interactive Jupyter notebook describing the analysis of the PM6 and Y6 NEXAFS, necessary transforms, and conversion to index of refraction is provided in our code repository.^64^

Although we focus here on Carbon K-edge indices of refraction, it is important to emphasize that other elemental K-edges may offer additional contrast opportunities. Unique heteroatom edges such as Nitrogen, Oxygen, or Sulfur K-edges may provide greater contrast, particularly if only one component contains the element. Further, if a heteroatom exists on a single moiety among many, then the orientation of that moiety might be independently measured. Some challenges exist for other K-edges. Nitrogen in particular can be challenging due to the ubiquitous use of thin silicon nitride membranes as a support substrate for films; the membrane itself significantly attenuates soft X-ray flux across the edge. Sulfur K-edge scattering occurs in an energy range often called “tender” X-rays. Although resonance-enhanced contrast tends to decrease with increasing atomic number of the elemental edge, the Sulfur K-edge nevertheless provides unique contrast modalities for organic electronics and other sulfur-containing materials.^65^

The absorptive β components across the Carbon K-edge (Figure 1e,f) reveal characteristic resonances for both materials. PM6 and Y6 each display a complex 1s → π* manifold containing multiple peaks arising from distinct conjugated structural elements, including fused rings and heterocyclic units, which exhibit different bond lengths and electronic environments. Both indices were extrapolated to maximize alignment of the 1s → π* manifold with the extraordinary direction. For Y6, this extrapolation results in near-complete extinction of 1s → π* resonances in the ordinary direction, indicating either coplanarity of the ring systems or a single dominant orientation moment within the reference film. In contrast, PM6 retains residual 1s → π* absorption in the ordinary direction even after maximal extrapolation (Figure 1e), suggesting a different orientation moment for a minority of π-conjugated subunits. Above the absorption edge (
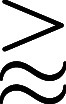
 291 eV), both molecules show stronger 1s → σ* intensity in the ordinary direction, consistent with the orientation of σ bonds described in Figure 1a,b. The dispersive components (Figure 1c,d), determined via Kramers–Kronig transformation, exhibit characteristic inflections near resonance peaks that can enhance RSoXS contrast even at energies offset from the absorption maxima. These material-specific δ(*E*) and β(*E*) functions provide the fundamental contrast necessary for probing phase separation in PM6:Y6 blends, while highlighting a key challenge: the dramatic differences between ordinary and extraordinary axes for each material imply that measured contrast will depend strongly on molecular orientation.

### Model Introduction

With the complex refractive indices established, we next develop structural models using the NIST RSoXS Simulation Suite (NRSS),^53,54^ a computational framework for simulating energy-dependent RSoXS patterns from real-space morphological representations. The NRSS is part of a growing family of SAS analysis methods, such as the Computational Reverse-Engineering Analysis for Scattering Experiments (CREASE) framework^66^ and others^67−69^ that use forward simulation from a detailed real-space representation to model and fit SAS data as an alternative to traditional approaches that separate form and structure factors and treat them with analytical expressions.^70^ The NRSS enables direct comparison between hypothesized morphological features and their corresponding scattering signatures by simulating the expected X-ray scattering through a voxelized representation that includes voxel-scale representations for molecular orientation direction and extent. Model construction requires careful consideration of typical features observed in OPV RSoXS data, particularly given the inherent interpretation challenges presented by the limited q-range accessible in RSoXS experiments. The RSoXS of OPV BHJs commonly exhibits a single, broad shoulder-like feature superimposed on a power-law background, a pattern that complicates unique structural assignment. Such features typically appear at *q*-values associated with real-space length scales commonly associated with BHJ phase separation, ranging from ≈10 to ≈200 nm. Such patterns have at least three possible structural interpretations, however, each with different implications for the BHJ morphology.

A first possible interpretation of the typical shoulder-like feature in OPV BHJ RSoXS would frame it as a Guinier-Porod-like^71^ crossover between power-law regimes. In this view, the characteristic *q*-value indicates an effective radius of gyration (*R*_g_)-like description of the average size of a minority phase. Notably, the low-*q* region in OPV RSoXS rarely plateaus to a truly flat Guinier regime, though with future construction of larger sample–detector distance RSoXS instruments, they may soon be routinely collected. A second possible interpretation considers the shoulder a broad and weak Bragg peak arising from regular center-to-center spacing between crystalline fibrils or lamellae, a phenomenon called a “long period" that is a regular occurrence in non-semiconducting semicrystalline polymers,^72^ and sometimes observed in semiconducting polymers.^73^ The third interpretation would frame the feature as subtle form factor oscillations. In this context, “form factor” scattering describes the scattering expected from a single object (fibril, sphere-shaped, etc.) and dependent only on its internal contrast distribution relative to its medium. Form factor scattering would potentially be broadened by a distribution of shape and size without any longer-range (e.g., center-to-center) correlations. In the form-factor interpretation, reliable size extraction would require observation of clear periodic minima in the scattering profile, which are rarely observed in OPV BHJ RSoXS. This fundamental ambiguity in RSoXS data interpretation necessitates careful consideration of model design to explore how different structural motifs manifest in RSoXS patterns and to establish which morphological features can be reliably distinguished through RSoXS analysis alone.

Given these possible interpretations, we must identify a model framework that makes minimal assumptions about the underlying morphological origins of the scattering features. There is limited experimental evidence for long-period ordering in donor–acceptor semiconducting polymers such as PM6. Similarly, the absence of clear form factor oscillations in typical RSoXS data precludes detailed analysis of discrete domain shapes. We therefore adopt a Gaussian Random Field (GRF) approach to construct our NRSS models, which provides a general framework for generating real-space composition descriptions from a specified power spectral density. The GRF algorithm employs random phase reconstruction to generate one possible real-space realization from among the infinite ensemble of composition descriptions that would produce the specified scattering pattern. GRFs have been used in other frameworks^74^ to understand complex scattering data. Although we will use a Guinier-Porod formalism (in the functional form proposed by Hammouda^71^) to represent the shape of the GRF power spectral density, this approach is particularly powerful because it remains agnostic to the physical origin of the scattering features — whether they arise from Guinier-Porod behavior, weak periodicity, or form factor contributions — while providing a computationally efficient means of generating realistic PM6:Y6 composition distributions. The GRF-generated composition descriptions serve as the foundation for exploring how variations in phase purity, molecular orientation, and surface roughness manifest in RSoXS patterns and in scattering intensity.

**Figure 2:**
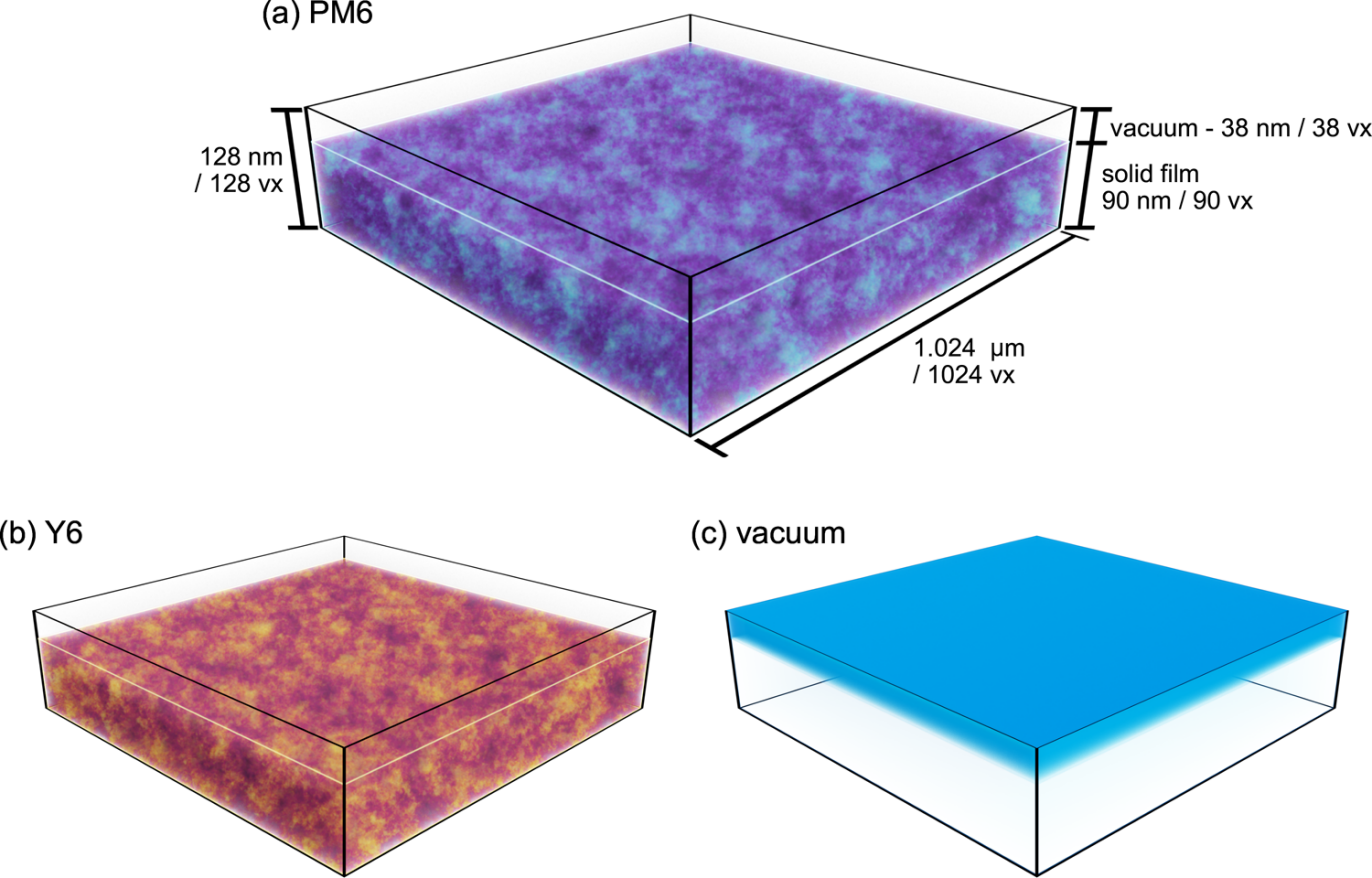
Three-dimensional representation of the NRSS model geometry for simulating RSoXS from PM6:Y6 blends. (a) Visualization of the PM6 component distribution within the model volume (1.024 μm × 1.024 μm × 128 nm, discretized into 1024 × 1024 × 128 voxels). The BHJ is 90 nm thick and bounded by 38 nm of vacuum, with the composition rendered in purple/cyan (cyan indicates higher volume fraction). (b) Complementary Y6 component distribution rendered in yellow/red (yellow indicates higher volume fraction), showing the phase-separated morphology. (c) Vacuum region above the film, rendered in blue, which is essential for incorporating surface roughness effects.

The complete NRSS model is shown in Figure 2. The composition description is generated using a GRF with a power spectral density supplied by a Guinier-Porod formalism with a low-*q* power law exponent of −0.5 to reflect the fact that a flat Guinier region is rarely seen in OPV BHJ RSoXS, and a high-q power law exponent of −3 that is roughly typical of OPV BHJ RSoXS, and an *R*_g_ transition in the mid-*q* range for a typical RSoXS experiment. The simulation volume consists of a 1.024 μm × 1.024 μm × 128 nm region discretized into 1024 × 1024 × 128 voxels, providing 1 nm resolution in all dimensions. Within this volume, we construct a 90 nm thick film (90 voxels) bounded by 38 nm of vacuum above, maintaining the total thickness of 128 nm. Three materials are present: PM6, Y6, and vacuum. The GRF-generated composition distribution assigns local volume fractions of PM6 (Figure 2a) and Y6 (Figure 2b) throughout the film volume, with complementary patterns reflecting phase separation between the two components. Each voxel contains a mixture of up to three materials, with their volume fractions determining the local complex refractive index. An explicit vacuum material (Figure 2c) enables the accurate modeling of surface roughness; although the initial model shown in Figure 2 is flat, we will later add surface roughness. Boundaries of this model are periodic in the lateral dimensions. This model construction allows us to systematically vary composition distributions, molecular orientations, and surface roughness while maintaining physically realistic optical properties throughout the simulation volume.

With our composition description established, we can now systematically evaluate the  analysis framework and phase purity under realistic experimental conditions. Although the indices of refraction shown in Figure 1 and the contrasts derived from them (Figures S1 and S2), provide the quantitative basis for most of the results, the NRSS framework allows us to (1) include realistic morphological scenarios where these contrasts are realized and combined, and (2) incorporate experimental limitations that affect real RSoXS measurements. By treating NRSS as a virtual instrument, we can directly account for experimental constraints such as the limited *q*-range accessible in typical RSoXS measurements and the restriction of detecting only in-plane scattering vectors at 90° incidence. The GRF-generated morphologies provide a controlled environment to investigate how realistic variations in molecular orientation and surface roughness might masquerade as changes in phase purity when interpreted through the conventional  analysis framework. This approach allows us to quantitatively assess the reliability of the  analysis framework under conditions that closely mirror actual experiments. Python code to recreate the models shown below is available upon request for those who wish to explore or extend this modeling framework.

### Phase Purity: Peak-to-Valley Composition Fluctuations

**Figure 3:**
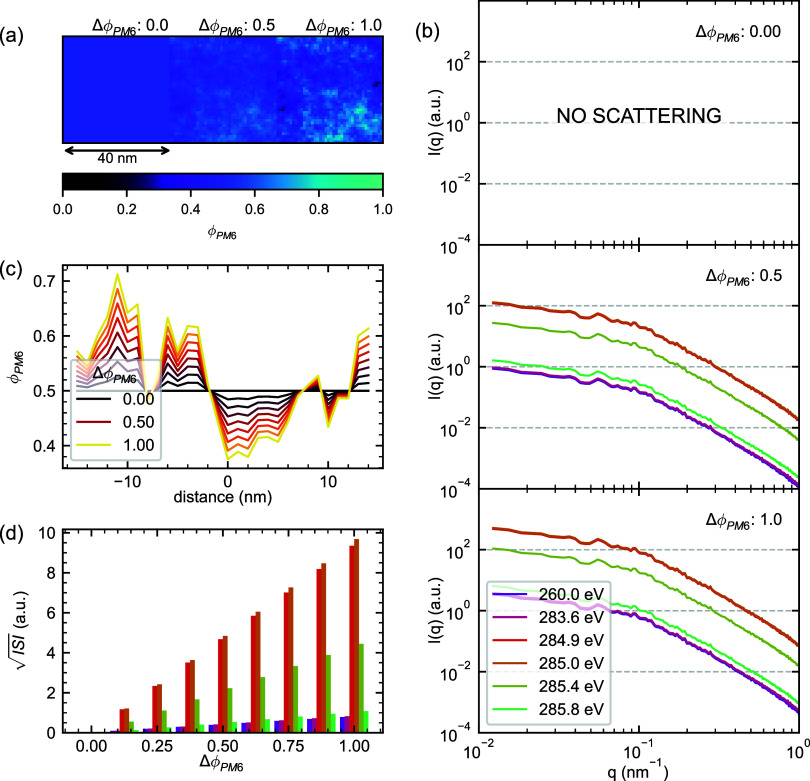
RSoXS patterns from model PM6:Y6 blends with varying phase purity. (a) Representative composition maps showing PM6 volume fraction (ϕ_PM6_) for three extents of phase separation (Δϕ_PM6_ = 0.0, 0.5, and 1.0). (b) Simulated RSoXS patterns *I*(*q*) at selected photon energies. (c) Line profiles extracted from the composition maps in (a), demonstrating how local PM6 content varies around the mean composition (ϕ_PM6_ = 0.5) with increasing phase separation amplitude. (d) Calculated  across the variation in Δϕ_PM6_ at different photon energies. The energy color legend from (b) also describes (d).

We begin by directly manipulating phase purity in the PM6:Y6 NRSS model to examine the central premise of the  analysis framework, starting with a simplified case having neither surface roughness nor molecular orientation in either component. Figure 3 presents a systematic investigation of phase purity effects using GRF-generated morphologies with increasing PM6-Y6 phase purity, controlled by the whole-model peak-to-valley PM6 composition amplitude Δϕ_PM6_. The Δϕ_PM6_ parameter ranges from 0 (completely mixed) to 1.0 (maximum phase separation), while maintaining a constant average composition of 50:50 PM6:Y6 throughout the film. By definition of the GRF approach, the voxel-by-voxel ϕ_PM6_ values follow a Gaussian distribution clustered around the 50:50 mean, with locally correlated excursions as shown in Figure 3a,c.

The simulated RSoXS patterns from this simplified parameter sweep provide an initial strong validation of the  analysis framework. When the system is completely mixed (Δϕ_PM6_ = 0), no significant scattering features appear, as expected for a single-phase system. As phase separation develops, the scattering patterns exhibit the Guinier-Porod behavior established by the GRF. Importantly, because the system contains only two phases, the patterns remain self-similar, varying only in relative intensity. Most significantly, Figure 3d demonstrates that , realistically integrated from *q* = 0.01 nm^–1^ to *q* = 1 nm^–1^, increases precisely linearly with phase purity under these idealized conditions. The relationship shows pronounced energy dependence, with scattering intensity variations stemming directly from the complex interplay between δ(*E*) and β(*E*) established in Figure 1, with maximum sensitivity to phase separation observed at ≈285.0 eV.

### Phase Purity: Interfacial Sharpness

Phase purity can manifest in a variety of ways. While the GRF algorithm naturally produces gradual composition variations, real BHJ systems could potentially exhibit significantly sharper interfaces. Such sharp compositional boundaries might arise from crystallization of one or both components, which can exclude the other phase, or from strong immiscibility between component liquids. To explore these effects, we modify our GRF-generated composition maps through an error function transformation that progressively sharpens the interfaces between PM6-rich and Y6-rich regions while maintaining the same characteristic correlation lengths and average composition. This transformation effectively pushes the system toward a more binary phase separation, allowing us to probe how interfacial sharpness influences RSoXS intensity distributions.

**Figure 4:**
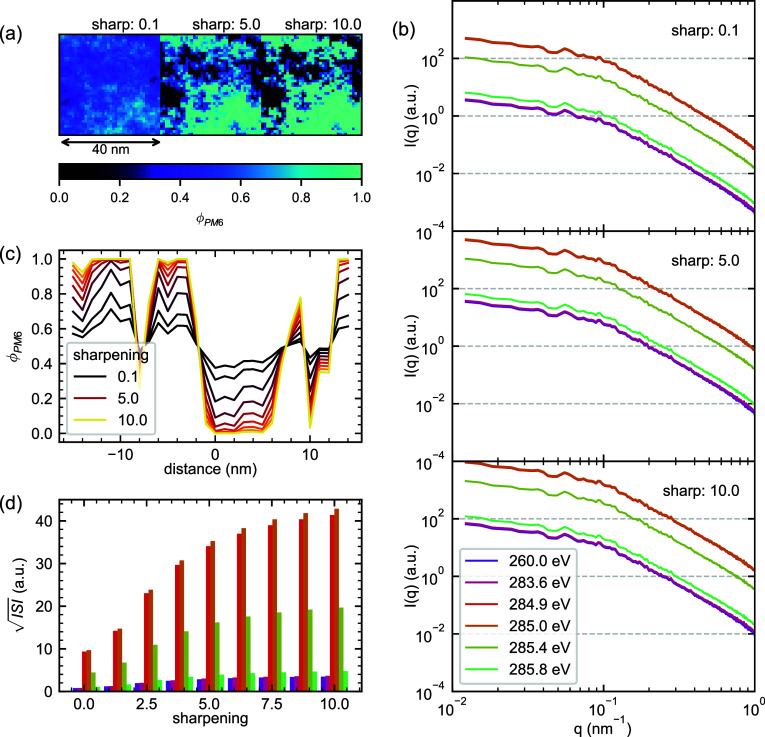
Impact of interfacial sharpening on RSoXS patterns from PM6:Y6 blends. (a) Composition maps showing PM6 volume fraction (ϕ_PM6_) for three extents of interface sharpening (0.1, 5.0, and 10.0) applied to a GRF morphology with Δϕ_PM6_ = 1.0. (b) Simulated RSoXS patterns *I*(*q*) at selected photon energies for each sharpening value, demonstrating enhanced scattering intensity with increased interfacial sharpness. (c) Line profiles through the composition maps, showing how sharpening drives local compositions toward binary extremes while preserving the underlying spatial correlations. (d) Evolution of  with increasing interfacial sharpness. The energy color legend from (b) also describes (d).

The effects of interface sharpening on RSoXS patterns are shown in Figure 4. Starting with a GRF-generated BHJ morphology with Δϕ_PM6_ = 1.0, we apply increasing extents of interface sharpening, transforming the initially gradual interfaces into more abrupt transitions between PM6-rich and Y6-rich regions. The transform was performed by multiplying a GRF composition field symmetric about zero with a factor, applying an error function, and then scaling the result to be bounded between ϕ_PM6_ of 0 and 1. The resultant morphology is thus always pure in the domain centers. At low scaling factors, the error function is essentially linear and thus the result exhibits the same compositional heterogeneity as the original GRF. At higher scaling factors, the input is compressed and therefore intermediate ϕ values in the GRF less than 0.5 are pushed closer to 0 and ϕ values greater than 0.5 are pushed closer to 1, resulting in a greater extent of phase purity and sharper interfaces. As shown in Figure 4a and quantified in the line profiles of Figure 4c, the sharpening parameter drives the local compositions toward the binary extremes of ϕ_PM6_ = 0 or 1, while maintaining roughly the same composition description imposed by the original GRF. The resulting RSoXS patterns (Figure 4b) maintain their characteristic Guinier-Porod appearance. As with our examination of Δϕ_PM6_ dependence, the model still has only two phases and therefore the patterns remain self-similar with energy-dependent intensity. The manifestation of interfacial sharpening on the  analysis framework is shown in Figure 4d, where  exhibits a strong dependence on the sharpening parameter. The nonlinearity of the dependence is reflective of the sharpening algorithm used. The variation in scattering intensity is larger here than in the Δϕ_PM6_ dependence because the overall phase purity is further increased with a starting point of Δϕ_PM6_ = 1 at the lowest sharpening condition. These results further demonstrate the effectiveness of the  analysis framework as a probe of phase purity in simple systems without surface roughness and without molecular orientation.

### Surface Roughness

Having examined how RSoXS responds to compositional heterogeneity in idealized flat films, we now turn to the influence of surface roughness on scattering patterns. Surface roughness is an inevitable feature of solution-processed BHJ films, arising from factors including solvent evaporation dynamics, substrate interactions, and phase separation processes such as crystallization. Readily characterized by atomic force microscopy (AFM) and generally considered a minor concern for GIWAXS measurements where periodic molecular packing dominates the signal, surface roughness can have profound effects on RSoXS patterns. This outsized influence stems from the high optical contrast between organic materials and vacuum (where β = 0, δ = 0), which exceeds the contrast between different organic phases at almost all photon energies (see Figure S1). The impact of surface roughness is particularly acute in RSoXS compared to other small-angle scattering techniques such as SAXS or SANS, where sample thicknesses are typically hundreds of microns up to several millimeters. In contrast, RSoXS samples are typically ≈100 nm thick, meaning height variations of even a few nanometers represent significant fractional sample volume. This combination of high vacuum contrast and exceptionally thin samples means that surface roughness can contribute substantially to the scattering intensity, potentially rivaling or exceeding the contributions from internal compositional heterogeneity that the  analysis framework aims to measure. To systematically investigate these effects, we modify our NRSS models to incorporate controlled extents of surface roughness while maintaining the underlying composition description previously established.

**Figure 5:**
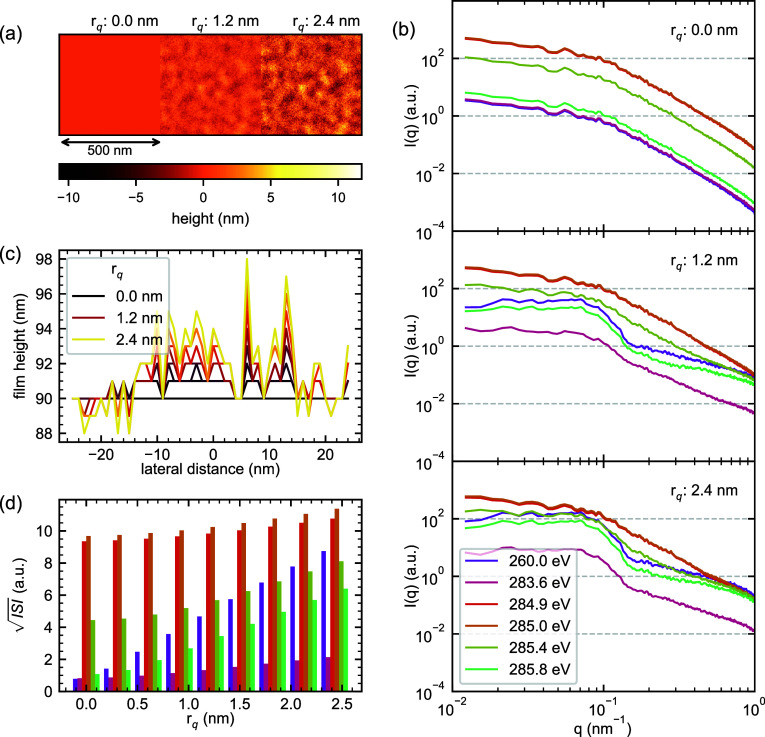
Impact of surface roughness on RSoXS patterns from PM6:Y6 blends. (a) Height maps showing surface topography for three extents of RMS roughness (*r*_q_ = (0.0, 1.2, and 2.4) nm) applied to a 90 nm thick film with a fixed composition description (Δϕ_PM6_ = 1.0). (b) Simulated RSoXS patterns *I*(*q*) at selected photon energies for each *r*_q_ value, demonstrating enhanced scattering intensity with increased surface roughness. (c) Height profiles extracted from the surface maps, showing the increasing amplitude of topographical variations with *r*_q_. (d) Evolution of  with increasing surface roughness at different photon energies, revealing substantial contributions to scattering intensity from surface roughness alone. The energy color legend from (b) also describes (d).

To systematically investigate surface roughness effects, we apply a GRF height field to the top surface of our model films. The height field was intentionally chosen to represent a “nodular” surface topography, which is a common result of AFM on OPV BHJ films. The GRF in this case has a peak in its power spectral density relating to a weak center-to-center correlation between adjacent nodules. The same GRF was used for each simulation with a different amplitude, and for each amplitude an effective root-mean-square (RMS) roughness (*r*_q_) was calculated following typical AFM practices. Figure 5a shows three simulated AFM topographies with *r*_q_ values of (0.0, 1.2, and 2.4) nm, a range of surface roughness values not uncommon in solution-processed BHJ films. For this analysis, we maintain a constant underlying composition description (Δϕ_PM6_ = 1.0) with no interfacial sharpening to isolate the effect of surface roughness variation alone.

The resulting RSoXS patterns, shown in Figure 5b, reveal that even modest surface roughness can dramatically influence scattering intensity and the shape of the scattering pattern itself. At *r*_q_ = 0, the patterns reflect only the underlying compositional heterogeneity. However, as *r*_q_ increases, we observe substantial enhancement of the scattered intensity across all measured *q* values. The patterns differ markedly depending on photon energy because there are now three phases present: PM6, Y6, and vacuum. Emergent pattern features at nonzero *r*_q_ are due to the nodular surface topography, not due to subsurface compositional heterogeneity. Some photon energies remain more sensitive to compositional heterogeneity, with a shape more similar to the original Guiner-Porod *I*(*q*) profile, and some photon energies are more sensitive to surface roughness effects, featuring an emergent peak-like feature, but the scattering pattern shapes and intensities at all energies are impacted to some extent by surface roughness. Figure 5d quantifies the impact of surface roughness on the  analysis framework, demonstrating that increasing surface roughness produces an energy-dependent intensity enhancement comparable to that observed from compositional heterogeneity. At many energies, the increase in  is larger with surface roughness than it is for any extrema of composition fluctuations. The relative magnitude of this enhancement varies significantly with photon energy, but in a pattern distinct from that observed for compositional contrast. The energy dependence underscores the importance of careful energy selection in the  analysis framework; even small differences in energy choice, such a 285.0 eV vs 285.4 eV, can be sufficient to dramatically alter the relative sensitivity to composition vs surface roughness.

Because the  analysis framework is frequently practiced on homologous series of OPV BHJ films that have different synthetic motifs or processing strategies, it would be natural to expect trends in surface roughness. This simulation campaign illustrates a real hazard that variations in  and features in *I*(*q*) might be interpreted as phase purity variations when they actually originate from differences in surface roughness. Best practices for this analysis framework should include ensuring that films are as smooth as possible and that members of a homologous film series have similar surface roughness. Careful analysis of the energy dependence of , which we will discuss later, can provide a means to differentiate between surface roughness and compositional effects in experimental data.

### Molecular Orientation: Voxel-by-Voxel Random “Orientation Noise”

Having demonstrated that realistic surface roughness can significantly influence the interpretation of the  analysis workflow, we now turn to an additional complicating factor in BHJ systems: molecular orientation. As demonstrated by the anisotropic optical constants in Figure 1, both PM6 and Y6 exhibit strong orientation-dependent interactions with X-rays near the carbon K-edge. While this molecular orientation sensitivity provides a powerful contrast mechanism unique to RSoXS, it also means that variations in local molecular orientation — whether correlated with composition or occurring independently — can contribute significantly to the scattering intensity. In organic semiconductors, orientational heterogeneity is extremely common, arising from texture development during crystallization, liquid-crystalline-like interactions that evolve during drying or heat treatments, and interfacial interactions at electrodes, substrates, and internal interfaces.

**Figure 6:**
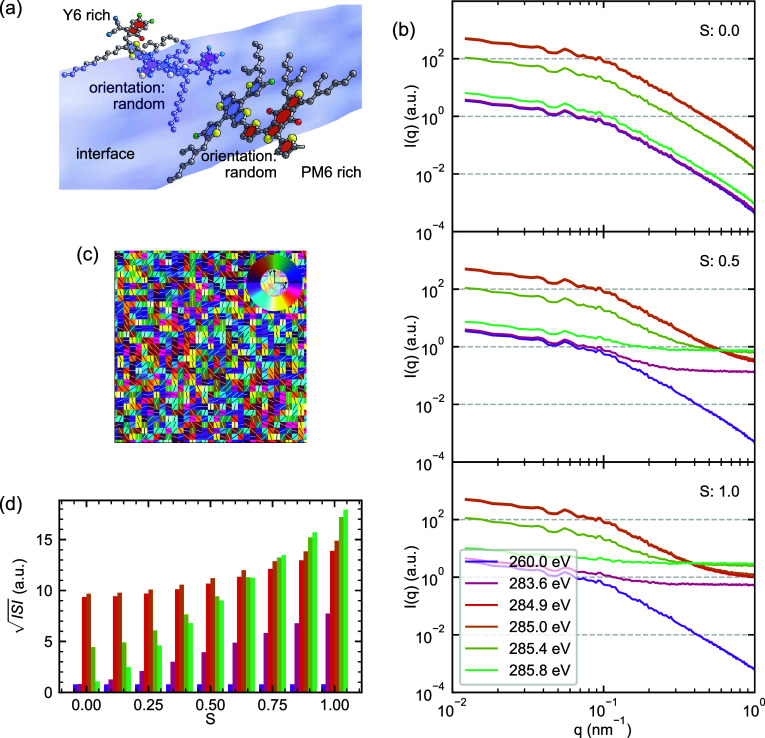
Impact of molecular “orientation noise” on RSoXS patterns. (a) Schematic illustration of the random orientation model, showing PM6 and Y6 molecules with orientations that vary independently of local composition. (b) Simulated RSoXS patterns *I*(*q*) at selected photon energies for increasing orientation extent (S). (c) Map of the in-plane projection of the extraordinary director, described by an Euler angle ψ. (d) Evolution of  with increasing orientation extent *S* at different photon energies, demonstrating substantial contribution to scattering intensity from orientational heterogeneity alone. The energy color legend from (b) also describes (d).

To begin exploring molecular orientation effects, we first consider a model in which molecular orientation varies randomly from voxel to voxel, independent of local composition. The idea that common orientation would occur at small length scales is particularly realistic as PM6 and Y6 molecules are large, such that our 1 nm^3^ voxel could accommodate only (very roughly) one molecule, and the likelihood of common orientation among the molecules would seem reasonably high. The idea that the molecular orientations could become truly randomized at the voxel length scale is less realistic given that intermolecular interactions would likely enforce a more gradual orientation distribution; this scenario is therefore provided as an informative extreme case. Figure 6a shows a cartoon schematic of internal interfaces within this model, on opposite sides of which the PM6 and Y6 molecules are randomly oriented. Both the out-of-plane Euler angle θ and the in-plane Euler angle ψ, which together describe the orientation of the extraordinary optical axis in three dimensions, were allowed to vary randomly. Figure 6c shows a map of the in-plane orientation of the extraordinary axis defined in Figure 1; in NRSS models this is described by a voxel-specific Euler angle ψ describing the in-plane projection of the extraordinary axis. Each model evaluation was performed with the same orientation map but different extents of orientation, described by an orientational order parameter *S*, which varies from 0 for isotropic molecules to 1 for perfect orientation parallel to voxel-specific extraordinary directors (such as those shown in Figure 6c). For simplicity in the exploration, a uniform S-value was applied to all voxels of both materials, though a more realistic model might include heterogeneity in the *S*-values as well.

The resulting RSoXS patterns, shown in Figure 6b, reveal that orientation noise alone can generate significant scattering intensity. As the parameter *S* increases from 0 (isotropic orientation) to 1.0 (completely oriented, randomly), we observe a systematic enhancement in scattered intensity across all measured *q* values and at all energies. In the scattering patterns, the effect of orientation noise is seen as a high-*q* upturn in the Porod slope. Concerningly, this effect appears very similar to the commonly observed fluorescence artifact in RSoXS, where the energy-dependent fluorescence from photon absorption events within the film adds an energy-dependent flat illumination field to each pattern. The energy dependence of orientation noise, however, is expected to be significantly different than that of fluorescence. The  analysis framework (Figure 6d) shows that this enhancement follows a distinct energy-dependent pattern compared to our previous investigations of compositional and surface roughness effects. At high orientation extents, new energies emerge to exhibit the strongest ISI (285.4 eV, 285.8 eV), primarily due to amplification of the high-*q* upturn by the *q*^2^ term of the ISI calculation; these energies are some of the least sensitive to compositional heterogeneity. The results once again underscore the challenge of energy selection in the  analysis framework: all energies are affected by orientation heterogeneity, and in this model scenario the energies with the strongest ISI are red herrings that are highly sensitive to orientation extent.

### Molecular Orientation: Interface-Directed Orientation

**Figure 7:**
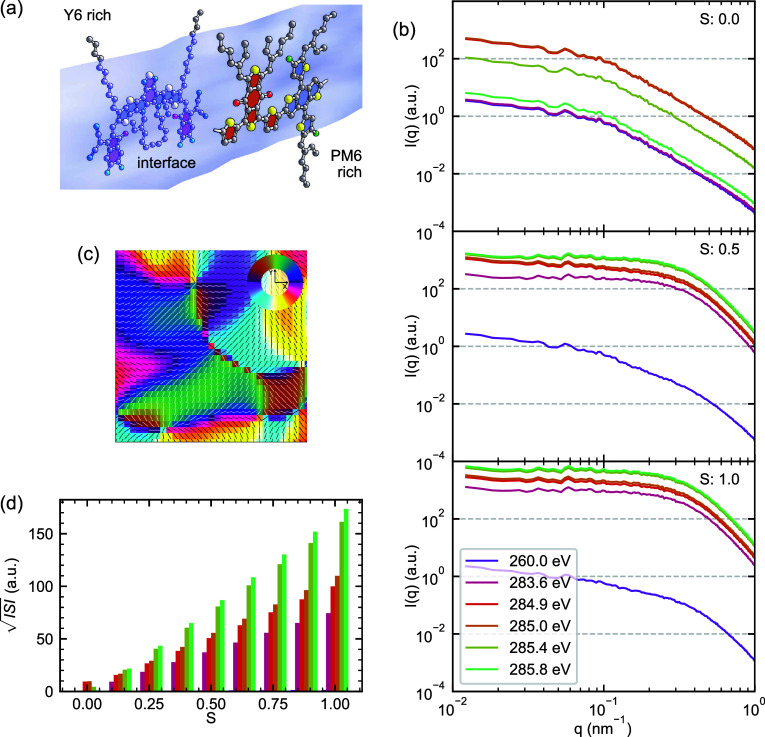
RSoXS patterns from PM6:Y6 blends with interface-directed molecular orientation. (a) Schematic illustration of molecular alignment at PM6-Y6 interfaces, where the extraordinary axes orient parallel to local composition gradients while maintaining edge-on texture relative to the substrate. (b) Simulated RSoXS patterns *I*(*q*) at selected photon energies for increasing orientation extent (*S*), showing distinct behavior from random molecular orientation. (c) Map of the in-plane projection of the extraordinary director, described by an Euler angle ψ. The associated composition field is shown as Figure S3. (d) Evolution of  with increasing molecular orientation extent *S* at different photon energies, demonstrating strong enhancement of scattering at energies typically associated with compositional contrast. The energy color legend from (b) also describes (d).

While random orientation provides a useful baseline for understanding how molecular orientation affects RSoXS patterns, real BHJ systems are additionally expected to exhibit orientational order correlated with compositional interfaces. The preferential alignment of conjugated molecules at donor–acceptor interfaces could arise from several physical mechanisms, including surface energy minimization or a preferential crystallization habit relative to a domain size and shape. To explore these effects, we present a model where the extraordinary axes of both PM6 and Y6 molecules align parallel to local composition gradients, effectively orienting the molecules “face-on” with respect to heterojunction interfaces while maintaining an overall edge-on molecular orientation relative to the substrate, as depicted in Figure 7a. This molecular orientation distribution was implemented by first applying Gaussian smoothing to the compositional map to suppress noise, then calculating local composition gradients to define the preferred molecular orientation direction at each point in space. The result is extremely reminiscent of liquid crystalline morphologies, as seen in Figure 7c, which describes the in-plane Euler angle (ψ) distribution; the out-of-plane Euler angle θ is fixed at 90° in this model to enforce edge-on orientation with respect to the substrate.

Figure 7 presents the results of this interface-directed orientation model. As with our random orientation model, we vary the extent of alignment through an order parameter *S*, but here *S* = 0 represents isotropic molecules while *S* = 1 indicates perfect alignment with the local composition gradient. The RSoXS patterns (Figure 7b) show dramatically different behavior compared to the random orientation case. As *S* increases, scattering intensity increases dramatically, and the *R*_g_-like feature in the Guinier-Porod profile is pushed out to higher *q*. Because this model has same Guinier-Porod composition description that we examined earlier, the change in feature location can only be explained by the addition of interface-directed orientation. More specifically, voxels that were once the origin of correlations in composition are now less correlated because each may have a different orientation; the orientation heterogeneity in a sense “breaks up” longer-range compositional correlations and pushes correlations to higher *q*. The  analysis framework (Figure 7d) reveals a complex energy dependence that differs markedly from both compositional contrast and random orientation scenarios. The energy dependence is different than that for the random orientation model because the molecular orientation here maintains an “edge-on” orientation relative to the substrate, whereas it was allowed to vary between edge-on and face-on in the random model. Strong scattering enhancement occurs at energies previously associated with compositional contrast (≈285.0 eV). Moreover, every energy is affected, and the dynamic range of  in this specific orientation scenario is more than 10× larger than that for pure composition variation (the *S* = 0 case).

### Molecular Orientation: Composition-Independent Orientation Correlation Length

**Figure 8:**
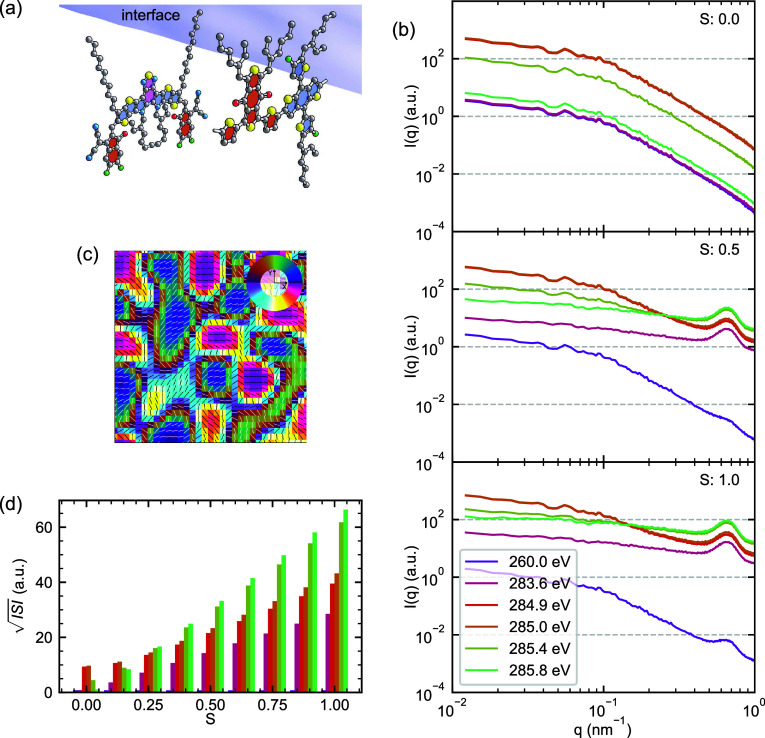
Impact of a composition-independent orientation correlation length on RSoXS patterns. (a) Schematic illustration showing how molecular orientation varies independently of the PM6-Y6 interface. (b) Simulated RSoXS patterns *I*(*q*) at selected photon energies for increasing orientation extent *S*, revealing emergence of new structural features. (c) Map of the extraordinary axis orientation, showing correlated domains with characteristic length scales smaller than the composition description. (d) Evolution of  with orientation extent *S*, demonstrating how orientational order can generate scattering features distinct from compositional heterogeneity. The energy color legend from (b) also describes (d).

After examining cases where molecular orientation is either random or directly coupled to compositional interfaces, we now investigate a model in which structured molecular orientation occurs independently of composition. Many factors during BHJ film formation including flow fields during coating, substrate interactions, and local crystallization events could potentially create orientational correlation lengths^75^ distinct from those governing compositional phase separation. To explore this possibility, we generated an orientation description using a GRF with a characteristic correlation length significantly shorter than that of our composition description. This orientation description determines the direction of the extraordinary axis at each point in space, completely decoupled from the local PM6:Y6 composition, as suggested in Figure 8a. The PM6 and Y6 molecules maintain their edge-on orientation relative to the substrate, but in-plane correlations of extraordinary axis orientation are now described by the highly correlated orientation description shown in the in-plane Euler angle ψ distribution of Figure 8c, in which can be seen a characteristic length scale significantly smaller than that of the interface-directed orientation description shown in Figure 7c. Once again, the out-of-plane Euler angle θ was kept at 90° to maintain edge-on orientation with respect to the substrate.

Figure 8 reveals how this independent orientation correlation length manifests in RSoXS patterns. At low *S* values, the scattering is dominated by compositional heterogeneity, showing the characteristic Guinier-Porod behavior observed in our previous models. However, as *S* increases, a narrow peak emerges in the scattering patterns at *q* ≈ 0.7 nm^–1^, corresponding to Bragg scattering from the orientation correlation length. These orientation-derived features become dominant, effectively masking contributions from the underlying composition heterogeneity. The emergence of these orientation-derived features is particularly evident in the high-q region, where the scattering intensity remains elevated compared to the power-law decay. The  analysis framework (Figure 8d) shows dramatic enhancement of scattering intensity with increasing *S*, with an energy dependence almost identical to that of the interface-coupled orientation model. This result demonstrates that structured molecular orientation alone can generate strong features in *I*(*q*) that could easily be misinterpreted as signatures of phase separation, particularly when an orientation correlation length falls within the q-range typically associated with BHJ domain sizes. The ability of structured molecular orientation to generate distinct spatial frequencies in RSoXS patterns, independent of composition, presents both a challenge for traditional interpretations and an opportunity for more sophisticated analysis methods that might distinguish between these contributions.

### Disentangling Morphological Contributions through Energy-Dependent Contrast

Our systematic investigation of composition, surface roughness, and molecular orientation effects reveals that the interpretation of RSoXS data through the  analysis framework is considerably more complex than traditionally assumed. These results prove incontrovertibly that there is no “safe” energy at which the  analysis framework can be employed to guarantee the results pertain only to composition variation. A single-energy analysis could easily mistake changes in  as composition effects when they actually stem from monotonic variations in surface roughness or differences in the extent or nature of molecular orientation heterogeneity within a homologous OPV BHJ series.

We contend that it is very likely that such misattributions of  trends are common in the published OPV BHJ RSoXS literature. And yet we acknowledge and celebrate the fact that RSoXS  trends still frequently correlate very well with trends in PCE and other device characteristics. We suspect this high degree of correlation is because surface roughness and molecular orientation heterogeneity, like compositional heterogeneity, are potentially strongly relevant to device performance. Increases in the crystallinity of one or more components, for example, would be expected to lead to increased surface roughness, increased orientation heterogeneity, *and* increased phase purity. In many cases, however, it may be valuable to separate the effects of each, to separately control and optimize different aspects of film morphology.

As we have noted throughout the sections of this manuscript, the variation of different aspects of BHJ morphology exhibit distinct energy-dependent scattering signatures. Although all mechanisms show some enhancement near the 1s → π* resonance (≈285 eV), their spectral shapes differ markedly. This variation arises from the fundamentally different ways each structural feature interacts with the X-ray beam: compositional heterogeneity relies on contrast between PM6 and Y6’s isotropic refractive indices (Figure S1), surface roughness engages contrast between organic solids and vacuum (Figure S1), and molecular orientation effects stem from the pronounced anisotropy in extraordinary and ordinary elements of the complex refractive indices (Figure S2). These distinct physical mechanisms manifest in different patterns of energy-dependent contrast enhancement, suggesting that careful analysis of  could provide a means to differentiate between various structural contributions.

**Figure 9:**
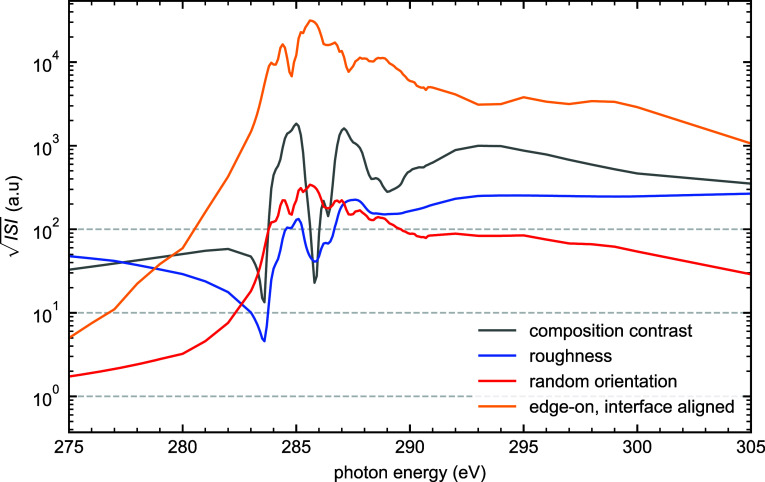
Energy-dependent  signatures across the carbon K-edge for four distinct morphological scenarios: compositional contrast (Δϕ_PM6_ = 1.0, gray), surface roughness (*r*_q_ = 2.4 nm, blue), random molecular orientation (*S* = 1.0, red), and interface-directed orientation (*S* = 1.0, orange). The distinct spectral shapes arise from different physical mechanisms of contrast generation, providing a potential basis for deconvolving multiple contributions to RSoXS patterns.

Figure 9 directly compares the energy-dependent  signatures across the carbon K-edge for four representative cases: pure compositional contrast (Δϕ_PM6_ = 1.0), surface roughness (*r*_q_ = 2.4 nm), random molecular orientation (*S* = 1.0), and interface-directed orientation (*S* = 1.0). Pure compositional contrast shows sharp features directly corresponding to the PM6 and Y6 NEXAFS resonances, with maximum intensity at 285.0 eV and lower intensity above the edge. Surface roughness, in contrast, exhibits broader spectral features and maintains significant intensity above 290 eV due to the β step edge contributions to vacuum contrast. The molecular orientation models present slightly different signatures, with the interface-aligned orientation scenario being much stronger due to its edge-on substrate-relative orientation.

Careful consideration of the energy-dependent  signatures leads us to propose these best practices in applying RSoXS to the measurement of OPV BHJs:

1. RSoXS measurements should collect data at multiple energies across resonances of interest with (0.1 to 0.2) eV increments. Single-energy experiments cannot distinguish between phase purity, surface roughness, and molecular orientation effects. For PM6:Y6, molecular orientation variations produce (10 to 100)× stronger signals than phase purity variations at most energies. All patterns should be corrected for artifacts that affect the apparent energy dependence such as fluorescence^52^ and absorption.^76^
2. High-quality NEXAFS spectra of individual components should be collected and analyzed before RSoXS experiments to develop refractive indices (Figure 1) and calculate contrast expectations (Figures S1 and S2). These calculations provide model-independent guidance for interpreting RSoXS data.
3. Strong scattering intensity above the absorption edge (
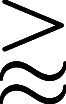
 291 for carbon) indicates significant Δβ contrast from elemental composition differences. This commonly arises from surface roughness (hydrocarbon-vacuum interfaces) but can also originate from other compositional heterogeneity: silicon nitride from common RSoXS substrates was implicated as the origin of certain features in a recent BHJ study, using a fully spectroscopic  analysis framework.^77^ In contrast, both compositional and orientational contrast between hydrocarbons typically show decreasing intensity above the edge.
4. Molecular orientation introduces a combinatorial increase in the number of possible contrasts (see Figure S2) and greatly increases the challenge of identifying the origin(s) of contrast. Although energy-dependence signatures might sometimes distinguish orientation from composition effects, forward simulation tools like the NRSS will often be necessary. NRSS models could be augmented with data fusion campaigns that incorporate information from other measurements, such as crystal orientation distributions from GIWAXS and surface relief information from AFM, to increase model uniqueness and verisimilitude and potentially extract more information from the RSoXS itself. Recent RSoXS characterization achievements in doped organic semiconductor systems^76^ illustrate the power of this approach.
5. When full spectroscopic analysis is impractical, results should acknowledge inherent ambiguity in the origin of scattering features. At minimum, surface roughness contributions should be ruled out. If clear identification of a specific origin of contrast is not possible, practitioners may describe contrast as arising from an unresolved combination of orientation and composition effects. Because orientation heterogeneity is likely relevant to BHJ operation, the information from an RSoXS measurement could remain useful despite an incomplete identification of its origin.

## Conclusions

Through systematic simulation of RSoXS patterns using the NIST RSoXS Simulation Suite, we have demonstrated that the conventional interpretation of  as a direct measure of phase purity in organic BHJs requires significant refinement. Although RSoXS remains a powerful probe of BHJ morphology, our results show that multiple aspects of morphology — including surface roughness, molecular orientation, and interfacial sharpness — can contribute to scattering intensity with magnitudes comparable to or exceeding those from compositional heterogeneity. The common practice of analyzing RSoXS data at a single energy, typically near the 1s → π* resonance, cannot distinguish between these contributions.

This finding does not invalidate the numerous studies that have found strong correlations between RSoXS  and device performance metrics. Rather, it suggests that these correlations may reflect a more complex relationship between morphological development and device operation than previously recognized. Processing steps that enhance phase separation often simultaneously affect surface roughness, molecular orientation, and interfacial structures. The sensitivity of RSoXS to this full gamut of morphological aspects may explain its robust correlation with device performance, even when the specific source of contrast enhancement has been misattributed.

Looking forward, our results point to opportunities for more sophisticated RSoXS analysis methods that leverage the complete energy-dependent scattering behavior. The distinct spectral signatures we have identified for different structural features suggest that careful analysis of energy-dependent contrast could enable deconvolution of multiple contributions to the scattered intensity. Forward simulation of RSoXS patterns, as demonstrated here, provides a powerful new approach for experimental design and data interpretation. By modeling the complete energy-dependent response of various structural features, the OPV community can identify optimal measurement energies for isolating specific morphological characteristics and predict spectral signatures that distinguish specific structural contributions. A simulation-guided approach, combined with careful analysis of complete carbon K-edge RSoXS spectra rather than measurements at isolated energies, enables more rigorous interpretation of experimental data. Future work combining these simulation capabilities with advanced data analysis methods and complementary characterization techniques promises to provide unprecedented insights into the complex relationships between processing, morphology, and performance in organic electronic materials.

## Supporting Information Available

Contrast calculation results for PM6 and Y6 including composition contrast, vacuum contrasts, and orientation contrasts (PDF)

## Footnotes

2 Certain commercial equipment, instruments, or materials are identified in this paper to foster understanding. Such identification does not imply recommendation or endorsement by the National Institute of Standards and Technology, nor does it imply that the materials or equipment identified are necessarily the best available for the purpose.
