# Supplementary material for: Resonant Soft X-ray Scattering for Organic Photovoltaics
Source: J Phys Chem B. 2025 Mar 26;129(13):3529–45. doi: 10.1021/acs.jpcb.5c00362 (PMC11973879; doi:10.1021/acs.jpcb.5c00362)
Supplement: Supplementary file 1 — jp5c00362_si_001.pdf [file jp5c00362_si_001.pdf]

Resonant Soft X-ray Scattering for Organic Photovoltaics

Dean M. DeLongchamp*

Materials Science and Engineering Division, National Institute of Standards and Technology, 100 Bureau Drive, Gaithersburg, MD 20899, USA

E-mail: dean.delongchamp@nist.gov

Contrast calculations

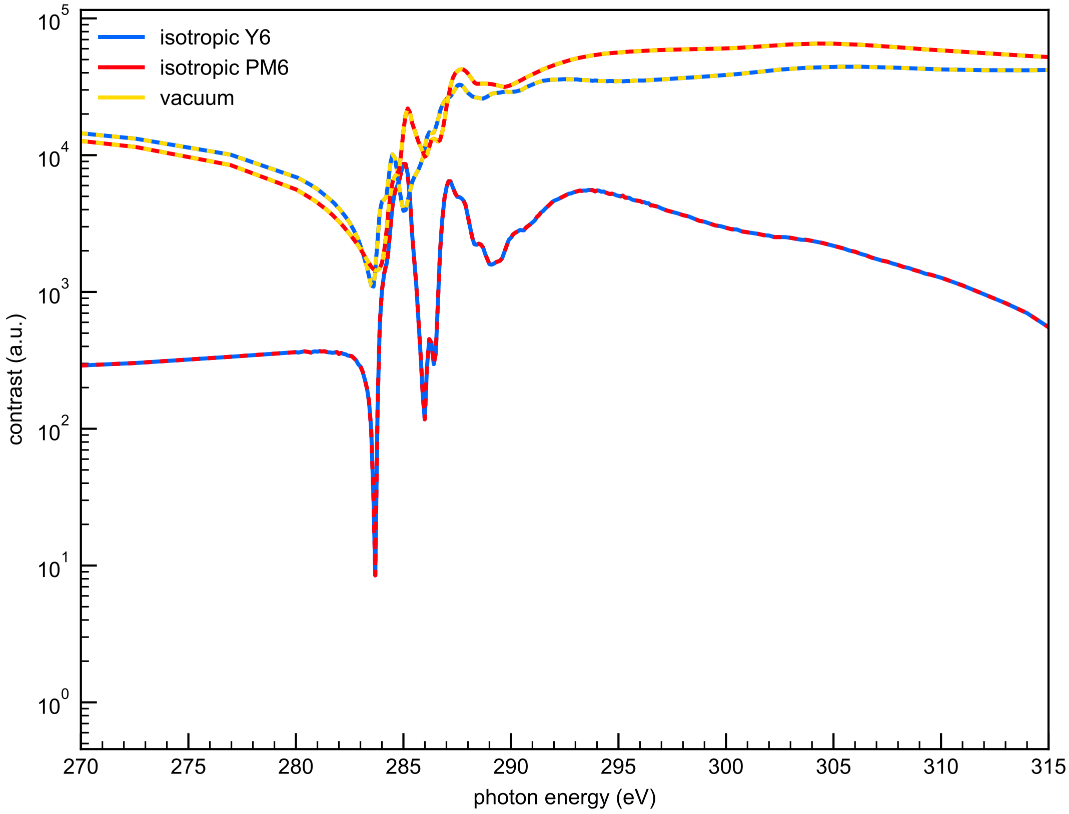

Figure S1: Energy-dependent scattering contrast across the carbon K-edge calculated from complex refractive indices. The solid lines show the contrast ($C(E) = E^4[\Delta\delta(E)^2 + \Delta\beta(E)^2]$) between isotropic PM6 and Y6 (red), between Y6 and vacuum (blue), and between PM6 and vacuum (yellow). Contrast is shown as a multicolored line including both colors of the binary pair of indices from which contrast originates. The vacuum contrast is consistently higher than the PM6-Y6 contrast across the entire energy range, highlighting why surface roughness can dominate RSoXS signals. Near the 1s$\rightarrow$ π^* resonance ($\approx$285 eV), sharp features in the PM6-Y6 contrast reflect the molecular absorption peaks, while the vacuum contrast shows broader features and remains elevated above the absorption edge due to the persistent step in β.

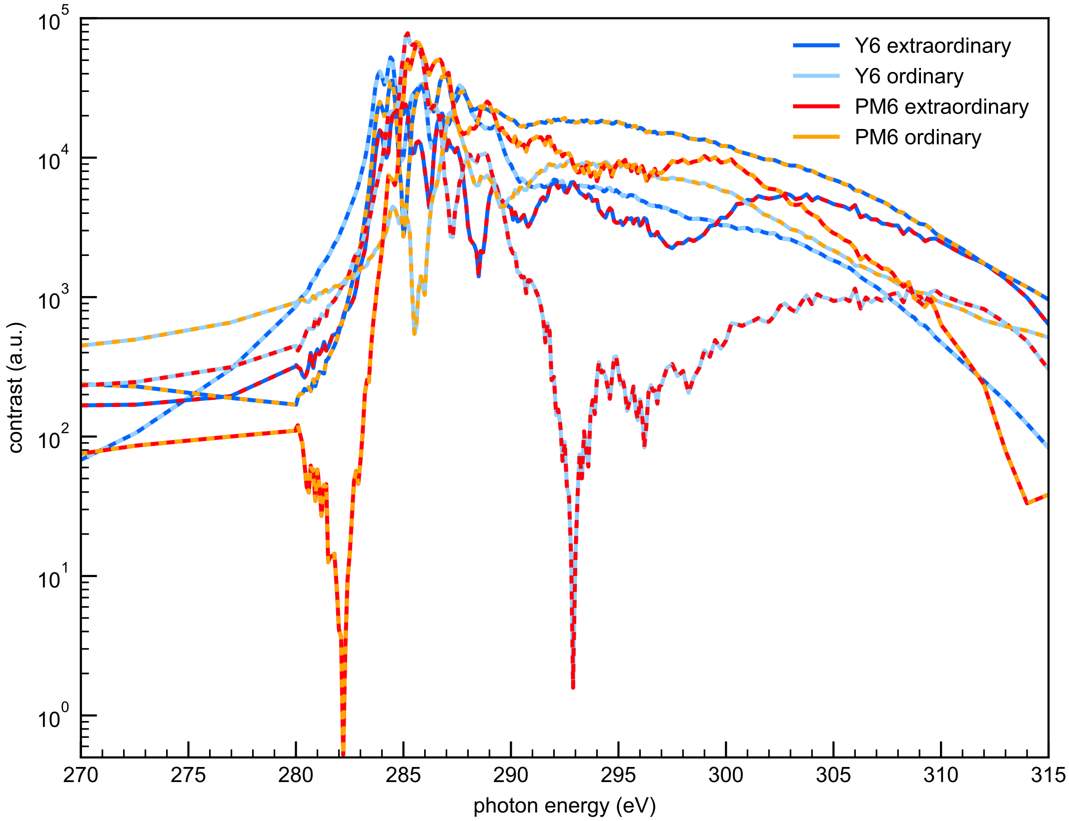

Figure S2: Energy-dependent orientational contrast across the carbon K-edge calculated from PM6 and Y6 anisotropic refractive indices. Contrast is calculated as $C(E) = E^4[\Delta\delta(E)^2 + \Delta\beta(E)^2]$ between each material's extraordinary and ordinary optical axes, representing the maximum possible orientation contrast. Contrast is shown as a multicolored line including both colors of the binary pair of indices from which contrast originates. Both PM6 (red/orange) and Y6 (dark/light blue) show strong dichroism near the 1s$\rightarrow \pi^*$ resonance (≈285 eV), with distinct patterns reflecting their different bond environments. These orientation-dependent differences in absorption provide an additional contrast mechanism in RSoXS that can eclipse compositional contrast in magnitude. The complexity in contrast outcomes described by this plot argues strongly for forward simulation as a means to predict and interpret RSoXS energy dependences.

Composition for interface-relative orientation

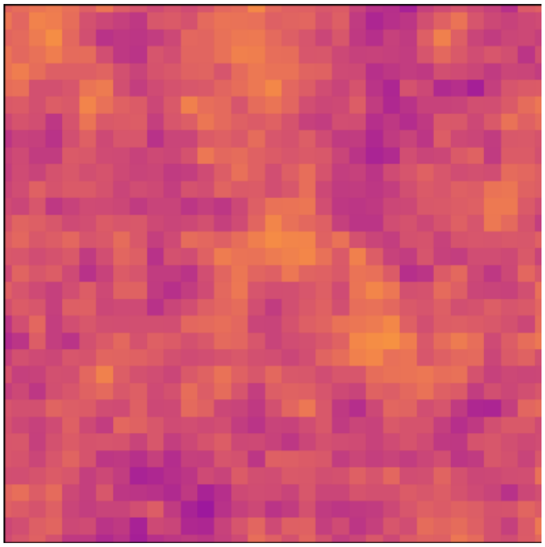

Figure S3: Compositional field associated with interface-relative orientation field shown in Figure 7c.
